# Sensory-Motor Neuropathy in Mfn2 T105M Knock-in Mice and Its Reversal by a Novel Piperine-Derived Mitofusin Activator[Fn fn4]

**DOI:** 10.1124/jpet.124.002258

**Published:** 2024-11

**Authors:** Jochen Weigele, Lihong Zhang, Antonietta Franco, Etienne Cartier, Gerald W. Dorn

**Affiliations:** Department of Internal Medicine (Pharmacogenomics), Washington University School of Medicine (J.W., L.Z., A.F., E.C., G.W.D.) and Mitochondria in Motion, Inc. (J.W., L.Z.), St. Louis Missouri

## Abstract

Mitochondrial dysfunction is a hallmark of many genetic neurodegenerative diseases, but therapeutic options to reverse mitochondrial dysfunction are limited. While recent studies support the possibility of improving mitochondrial fusion/fission dynamics and motility to correct mitochondrial dysfunction and resulting neurodegeneration in Charcot-Marie-Tooth disease (CMT) and other neuropathies, the clinical utility of reported compounds and relevance of preclinical models are uncertain. Here, we describe motor and sensory neuron dysfunction characteristic of clinical CMT type 2 A in a CRISPR/Casp-engineered Mfn2 Thr105Met (T105M) mutant knock-in mouse. We further demonstrate that daily oral treatment with a novel mitofusin activator derived from the natural product piperine can reverse these neurologic phenotypes. Piperine derivative 8015 promoted mitochondrial fusion and motility in Mfn2-deficient cells in a mitofusin-dependent manner and reversed mitochondrial dysfunction in cultured fibroblasts and reprogrammed motor neurons from a human CMT2A patient carrying the MFN2 T105M mutation. Like previous mitofusin activators, 8015 exhibited stereospecific functionality, but the more active stereoisomer, 8015-P2, is unique in that it has subnanomolar potency and undergoes entero-hepatic recirculation which extends its in vivo half-life. Daily administration of 8015-P2 to Mfn2 T105M knock-in mice for 6 weeks normalized neuromuscular and sensory dysfunction and corrected histological/ultrastructural neurodegeneration and neurogenic myoatrophy. These studies describe a more clinically relevant mouse model of CMT2A and an improved mitofusin activator derived from piperine. We posit that 8015-P2 and other piperine derivatives may benefit CMT2A or other neurodegenerative conditions wherein mitochondrial dysdynamism plays a contributory role.

**SIGNIFICANCE STATEMENT:**

Mitochondrial dysfunction is widespread and broadly contributory in neurodegeneration, but difficult to target therapeutically. Here, we describe 8015-P2, a new small molecule mitofusin activator with ∼10-fold greater potency and improved in vivo pharmacokinetics versus comparators, and demonstrate its rapid reversal of sensory and motor neuron dysfunction in an Mfn2 T105M knock-in mouse model of Charcot-Marie-Tooth disease type 2 A. These findings further support the therapeutic approach of targeting mitochondrial dysdynamism in neurodegeneration.

## Introduction

Dysfunction of mitochondria wherein oxidative phosphorylation produces ATP that fuels most cellular processes in multicellular organisms is increasingly implicated in human disease. Thus, damage of the mitochondrial genome causes Kearns-Sayre syndrome, mitochondrial myopathy, encephalopathy, lactic acidosis, and stroke-like episodes syndrome, neurogenic weakness, ataxia and retinitis pigmentosa, and Leber hereditary optic neuropathy, all of which are notable for neurologic involvement (Taylor and Turnbull, 2005). A more limited form of mitochondrial pathology is produced by impaired mitochondrial dynamism, typically defined as mitochondrial fusion, fission, and motility. Mitochondrial dysdynamism is observed in, and posited to contribute to, multiple neurodegenerative diseases (De Vos et al., 2007; Knott et al., 2008; Chen and Chan, 2009; Sheng and Cai, 2012; Chen et al., 2014; Dang et al., 2023), but is unambiguously the underlying cause of the sensory-motor neuropathy, Charcot Marie Tooth (CMT) type 2A, provoked by mutations of mitofusin (MFN) 2 (Züchner et al., 2004; Dorn, 2020a, 2021; McCray and Scherer, 2021). CMT2A has been associated with approximately 100 different MFN2 mutations, predominantly evoking diminished or complete loss-of-function (Larrea et al., 2019). In most instances, CMT2A manifests as delayed neuromuscular development in childhood with progressive deterioration in neuromuscular function through adolescence and stabilization thereafter. Although the disease affects both sensory and motor neurons, motor neuropathy tends to be more pronounced than sensory dysfunction (Feely et al., 2011; Fridman et al., 2015; Stuppia et al., 2015; Pipis et al., 2020). The majority of confirmed CMT2A patients have mutations within the MFN2 GTPase domain presumed to impair catalytic GTPase activity essential to mitochondrial fusion (although actual biochemical GTPase inactivity has only been demonstrated in select GTPase domain mutations). Thus, CMT2A is not only an incurable and currently untreatable debilitating hereditary neuropathy, but an experiment of nature that provides a clinically relevant platform in which the functional importance of mitofusin in mitochondrial dynamism can be interrogated in relation to integrated neuromuscular function.

MFN2 is one of two nuclear-encoded mitofusin proteins that promote mitochondrial fusion and motility (Chen and Chan, 2010; Dorn, 2020b). Compared with MFN1, MFN2 may have additional unique roles in mitochondrial quality control through the PINK-Parkin mitophagy pathway (Shirihai et al., 2015; Joaquim and Escobar-Henriques, 2020) and mitochondrial-endoplasmic reticulum calcium crosstalk (Naon and Scorrano, 2014; Mao et al., 2022; Zaman and Shutt, 2022; Naón et al., 2023). One of the validated MFN2 GTPase-inactivating CMT2A mutations is MFN2 T105M (Franco et al., 2022). MFN2 T105M, either expressed using adenoviral vectors in mouse embryonic fibroblasts or in the context of a heterozygous mutation in dermal fibroblasts from a human CMT2A patient, inhibits normal mitochondrial fusion, manifested as mitochondrial fragmentation, depolarization, and clumping (Detmer and Chan, 2007; Dang et al., 2022). In mouse and human neuronal axons, MFN2 T105M provokes mitochondrial fragmentation and clumping while additionally suppressing mitochondrial motility (Rocha et al., 2018; Franco et al., 2020, 2022, 2023). Transgenic overexpression of MFN2 T105M in mice using neuron-specific promoters reportedly changed the structure and reduced the number and motility of axonal mitochondria while altering skeletal muscle fiber size and distribution. Likewise, forced neuronal Mfn2 T105M expression provoked neuromuscular phenotypes such as hindlimb and gait abnormalities, decreased Rotarod latency, and diminished compound muscle action potential measured by neuroelectrophysiological testing (Detmer et al., 2008; Bannerman et al., 2016; Franco et al., 2020, 2022).

Recent studies have revealed that allosteric mitofusin activation can reverse neuromuscular dysfunction in the Mfn2 T105M motor neuron transgenic mouse model of CMT2A (Franco et al., 2020, 2022). These reports describe mitigating effects of two phenylhexanamide class mitofusin activators (Dang et al., 2020; Dang et al., 2021). However, applicability of this forced transgenic expression CMT2A model to the human condition remains uncertain because of abnormally high expression of the mutant MFN2 transgene in neurons and absence of normal *Mfn* transcriptional control mechanisms. Moreover, because MFN2 T105M in this mouse model is expressed exclusively in motor neurons it has not been possible to evaluate sensory defects characteristic of clinical CMT2A.

Here, we describe a mouse model of CMT2A in which the MFN2 T105M mutation was gene-edited into the highly homologous endogenous *Mfn2* mouse gene. This “knock-in” (KI) approach expresses the mutant protein in all tissues and preserves natural regulatory mechanisms. Using this KI mouse line, we evaluated CMT2A-mitigating effects of a novel piperine-derived mitofusin activator of the “reverse carboxamide” class (Zhang et al., 2022), designated 8015-P2. Our results show that motor and sensory abnormalities of Mfn2 T105M KI mice are rapidly reversed by daily oral administration of 8015-P2.

## Materials and Methods

### Mouse Lines.

All experimental procedures were approved by the Washington University in St. Louis School of Medicine Animal Studies Committee, International Animal Care and Use Committee protocol numbers 22-0314 and 23-0025. Mice were housed on a 12-hour light/dark cycle with ad libitum access to food. Neuromuscular and sensory phenotype evaluations were carried out in a separate study room after acclimatization for at least 20 minutes. Mfn2 T105M motor neuron transgenic mice have been previously reported and extensively characterized (Franco et al., 2020, 2022). Briefly, flox-STOP-hMFN Thr105Met mice (C57BL/6 Gt(ROSA)26 Sortm1 (CAG-MFN2*T105M)Dple/J; The Jackson Laboratory, Bar Harbor, ME #025322) were crossed to mice expressing Cre recombinase under control of the transcription factor Mnx1-HB9 (B6.129S1-Mnx1tm4(cre)Tmj/J; The Jackson Laboratory #006600). Mice carrying both gene constructs express MFN2 T105M in motor neurons beginning in utero.

The creation of mice with human CMT2A mutations MFN2 T105M and M376V CRISPR-engineered into the mouse Mfn2 gene has been reported, as was absence of a cardiac phenotype (Franco et al., 2023). Neuromuscular phenotyping was performed for the current studies (vide infra). Results of male and female mice were combined as there were no observed sex differences.

Mice with lox-P flanked *Mfn1* or *Mfn2* alleles were purchased from Jackson Laboratories (strain # 026401 and 026525, respectively) and interbred to generate Mfn1/Mfn2 double floxed (*Mfn1* fl/fl/*Mfn2* fl/fl) mice as a source of dorsal root ganglion neurons (DRG) in which expression of one or both mitofusins could be abrogated using adeno-Cre.

### Cultured Cells.

Mfn1 null, Mfn2 null, and Mfn1/Mfn2 double null mouse embryonic fibroblasts (MEFs) were purchased from American Type Culture Collection (ATCC Manassas, Virginia, Cat#: CRL-2992, CRL-2994 and CRL-2993, respectively) and cultured under standard conditions.

DRG neurons were isolated from ∼8-week-old Mfn2 T105M motor neuron transgenic mice, Mfn2 T105M KI mice, or *Mfn1* fl/fl/*Mfn2* fl/fl mice and cultured as described (Dang et al., 2023).

Primary human dermal fibroblasts from a CMT2A patient carrying the MFN2 T105M mutation, obtained under an Institutional Review Board-approved protocol in accordance with the Declaration of Helsinki and generously provided by Dr. Robert H. Baloh (Cedars-Sinai, Los Angeles CA, USA), were metabolically stressed by replacement of glucose in the tissue culture medium with galactose (Dang et al., 2022) prior to evaluation of mitochondrial aspect ratio and polarization status. These human CMT2A fibroblasts were also directly reprogrammed into human CMT2A motor neurons using microRNA-mediated conversion exactly as described (Abernathy et al., 2017; Franco et al., 2020).

### Viral Vectors.

MFN2 wild-type, MFN2 CMT2A mutant, and MFN2 Forster resonance energy transfer (FRET) viral constructs were engineered at Vector Biolabs and have previously been reported (Franco et al., 2020, 2023). Cells were transduced at a multiplicity of infection of 50 (MEFs) or 100 (dorsal root ganglion neurons). Other viral vectors were purchased: adenovirus *β*-galactosidase (Vector Biolabs Cat#: 1080), adenovirus Mito-Ds-Red2 (Signagen Cat#: 12259) and adenovirus Cre-recombinase (Vector Biolabs Cat#: 1794).

### Antibodies and Stains.

Mouse monoclonal anti-MFN1 and anti-MFN2 were from AbCAM (Cat#: ab126575 and ab56889; 1:1000 dilution in AbCAM 10X Blocking Buffer Cat#: ab126587), rabbit polyclonal anticytochrome c oxidase subunit 4 (COX-IV) was from AbCAM (Cat#: ab16056; 1:1000 dilution), mouse monoclonal antiglyceraldehyde-3-phosphate dehydrogenase was from AbCAM (Cat#: ab8245; 1:3000 dilution), *α*-bungarotoxin Alexa-Fluor 594 was from ThermoFisher (Cat#: B12423; 0.5 *μ*g/ml), Alexa-Fluor 488 goat antirabbit was from ThermoFisher (Cat#: A11008; 1:400 dilution), fluorescein-conjugated wheat germ agglutinin was from Invitrogen (Cat#: W834; 1:50 dilution), MitoTracker Orange was from Thermo Fisher (Cat#: M7510), tetramethylrhodamine ethyl ester was from Thermo Fisher (Cat#: T669), and Hoechst nuclear stain was from Thermo Fisher (Cat#: H3570). Immunoblotting and staining for confocal microscopy used standard techniques as previously described (Franco et al., 2020).

### Mitofusin Activators.

*N-(trans-4-hydroxycyclohexyl)-6-phenylhexanamide (trans*-MiM111) [designated Cpd 13B in (Dang et al., 2020) or MiM-111 in (Franco et al., 2020) and (Franco et al., 2022)] was used to establish full fusogenicity in dose-response curves. Compound 8015 and its enantiomers 8015-P1 and 8015-P2 (Dorn, 2023) were synthesized at WuXi Apptech Co., Ltd. (Shanghai, China). All mitofusin activators were obtained from Mitochondria in Motion, Inc. (Saint Louis MO, USA) under terms of a Material Transfer Agreement. Aliquots of all mitofusin activators were initially dissolved in DMSO as 10 mM stocks at -20°C. For in vivo oral administration in CMT2A mouse models, 8015 and 8015-P2 were diluted 1:100 or greater into a vehicle of 70% water/30% 2-hydroxypropyl-*β*-cyclodextrin (Sigma Cat#: 332607).

Routine in vitro pharmacokinetic (PK) profiling of 8015 and its enantiomers (8015-P1 and 8015-P2) was performed at WuXi Apptec Co., Ltd. (Shanghai, China) using standard methods as described (Dang et al., 2020, 2021). In vivo PK profiling was performed by Frontage Laboratories (Exton PA, USA). The vehicle used for intravenous and oral PK studies was 12% sulfobutylether-*β*-cyclodextrin (SBE-*β*-CD; CyDex Pharmaceutical Inc., Lenexa KS, USA) in sterile water.

Enterohepatic recirculation (EHR) of 8015-P2 was tested by fasting mice overnight prior to administration of the compound (5 mg/kg) by oral gavage. 1 hour later, activated charcoal (2 g/kg) was administered by oral gavage to half of the mice; all mice were fed 2 hours after 8015-P2 dosing to induce gall bladder emptying. Plasma 8015-P2 levels were assayed using LC/MS (Dorn, 2023) at eight time points (0.25–12 h) after administration and compared between mice that did or did not receive activated charcoal. Areas under the concentration curves were calculated from the raw data; plasma half-times were estimated by linear regression of the second, slower elimination phase.

### Live Cell Imaging of Mitochondria in Cultured Cells and Sciatic Nerve Axons.

Mitochondrial polarization based on tetramethyl rhodamine ester fluorescence and fusogenicity based on an increase in mitochondrial aspect ratio were measured in static confocal images of live cells using methods that we have extensively validated (Franco et al., 2016, 2020; Rocha et al., 2018; Zhang et al., 2022). As necessary, red, green, and blue brightness, or rarely contrast, were adjusted in individual confocal images prior to merging to enhance clarity. All adjustments were performed across the entire image.

Mitochondrial elongation was assessed in Mfn2 null MEF cells after treatment with compounds at concentrations ranging from 0.5 nM–10 *μ*M in DMSO for 14 h. Mitochondria were stained with MitoTracker Orange, and nuclei were costained with Hoechst before confocal microscope imaging; at least three independent experiments were performed. Mitochondrial aspect ratio (length/width) of at least 10 cells was measured for each experiment using National Institutes of Health ImageJ. Compound fusogenicity was quantified as mitochondrial aspect ratio and indexed to the maximal response evoked by the mitofusin activator trans-MiM111. Concentration-response curves were generated using a sigmoidal model in Prism 8 software. EC50 and Emax values and their variances were calculated from interpolated data of all replicate experiments in a series.

Mitochondrial motility in neuronal processes of cultured reprogrammed human motor neurons (Abernathy et al., 2017; Dang et al., 2023), mouse DRG neurons transduced with adeno-Mito-DS-Red2, or axons of ex vivo mouse sciatic nerves stained with tetramethyl rhodamine ester was measured using time-lapse confocal microscopy on a Nikon Eclipse T*i*2 confocal microscope as described (Franco et al., 2016, 2020; Rocha et al., 2018). Mouse DRG neurons and reprogrammed human CMT2A motor neurons were transduced with adeno-Mito-DS-Red2 to visualize mitochondria; ex vivo sciatic nerve mitochondria were labeled with tetramethyl rhodamine ester (200 nM for 30 minutes at 37° C). Time-lapse imaging was carried out using confocal microscopy on a Nikon Eclipse Ti2 confocal microscope with 1 frame captured every 5 seconds for 180 frames (15 minutes). Kymographs and quantitative data were generated using the ImageJ plug-in: Velocity_Measurement_Tool.

FRET of wild-type and T105M MFN2 tagged at the amino terminus with Cerulean- and the carboxyl terminus with Venus-fluorescent proteins was performed on mitochondria isolated from Mfn1/Mfn2 double knockout MEFs expressing those viral constructs (50 multiplicity of infection for 48 hours) in a 96-well format as described (Dang et al., 2020). FRET signals were acquired on a Tecan Safire II multimode plate reader: FRET – Excitation 433/8 nm, Emission 528/8 nm; Cerulean – Excitation 433/8 nm, Emission – 475/8 nm, and were normalized to the Cerulean signal.

### Evaluation of Mouse Neuromuscular Phenotypes.

Mfn2 T105M knock-in mice underwent evaluation at 10-week intervals to establish basal phenotype progression. 8015 or 8015-P2 treatment studies were performed after final baseline evaluations at 50 weeks of age. 8015 (transgenic MFN2 T105M model) or 8015-P2 (Mfn2 T105M knock-in model) were administered at doses of 50 mg/kg by oral gave once daily in the morning. Repeat phenotyping was performed after 4 and 8 weeks of treatment (transgenic model) or 6 weeks of treatment (knock-in model), after which the mice were killed by anesthesia overdose and tissue specimens obtained for histologic and ultrastructural studies, as described (Franco et al., 2020).

The protocols used for Rotarod and neuro-electrophysiology testing of sciatic nerve/tibialis muscle function (in transgenic CMT2A and ALS mice) have been published (Franco et al., 2020, 2022; Dang et al., 2023) and are briefly described here:

*Rotarod studies* were performed on mice initially familiarized and trained on the Rotarod (Ugo Basile, Gemonio, VA, Italy;# 47650) at a speed of 5 rpm. For evaluation, the initial speed of 5 rpm accelerated to 40 rpm over 120 seconds and then maintained 40 rpm for a maximum of 180 seconds. For each trial, five tests were performed with at least 5 minutes rest between each test. No trends were observed from test 1 to test 5. In total, three trials were performed over sequential days to account for variabilities of the Rotarod test and between days. For each mouse, the latency (time to falling off) of replicate tests for each trial day were averaged, and results of the three independent trials were averaged to obtain a result for that condition (i.e., age, genotype, and/or drug treatment).

*Neuroelectrophysiologic recordings* of hindlimb compound muscle action potentials (CMAP) were performed with a Viasys Healthcare Nicolet Biomedical instrument (Middleton, WI, USA Cat:# OL060954) using Viking Quest version 11.2 software. Nerve stimulation used 3.9 mV pulses of 0.002 milliseconds duration administered through a needle electrode placed at the proximal sciatic nerve through a posterior lumbar approach. Muscle depolarization was monitored using a loop electrode placed around the mid distal hindlimb, corresponding to the greatest girth of the tibialis and gastrocnemius muscles.

An *inverted grid suspension test* was used to assess grip strength and coordination. Mice were placed on a grid, which was slowly inverted no higher than three feet above a padded surface; the time to falling off (latency) was recorded up to a maximum of 500 seconds. Each mouse underwent at least three independent studies on different days, and the longest latency time was used (Aartsma-Rus and van Putten, 2014). Obese mice (>150% of mean weight for that condition) were preemptively excluded.

Because Mfn2 T105M knock-in mice express mutant Mfn2 systemically, the battery of neurologic tests for these animals was expanded to include sensory nerve testing:

*Sensitivity to a thermal stimulus* was measured using tail-immersion in a warm water bath (46°C) until tail withdrawal (flicking response) or other adverse reactions were observed (Courteix et al., 1993; Petit et al., 2014). Each mouse underwent at least three trials per condition and the data were averaged.

*Mechanical sensitivity* was measured using the von Frey test (Martinov et al., 2013). Mice were placed in a plexiglass chamber with a mesh grid floor and allowed to accommodate for at least 20 minutes or until exploratory behavior had ceased. The response to mechanical stimulation of hind paws used IITC Supertips filaments and an electronic von Frey anesthesiometer (IITC Life Science, Inc. Woodland Hills CA, USA). Filaments were applied to the footpads with a gradual increase in pressure until paw withdrawal was evoked; the maximum applied force was recorded. Paws of the left and right foot were alternately measured, with at least six measurements taken per animal and the results averaged for each mouse.

### Tissue Histology and Ultrastructure.

Confocal imaging of gastrocnemius myocyte cross-sectional area (wheat germ agglutinin staining) and synapse density (*α*-bungarotoxin staining) was performed as described (Franco et al., 2020). Briefly, gastrocnemius muscles were fixed in 4% paraformaldehyde, transferred to 30% sucrose/phosphate buffered saline overnight at 4°C, and embedded in optimal cutting temperature medium (Tissue-TEK Cat: 4583). At least 80 wheat germ agglutinin-stained myocytes were measured per mouse and their areas calculated using National Institutes of Health ImageJ. For mitochondrial occupancy in neuromuscular synaptic junctions, 10 *μ*m cryostat sections were stained using anti-COXIV (1:200 in 10% goat serum) and synapses labeled with *α*-Bungarotoxin (0.5 *μ*g/ml in 10% goat serum). At least four separate microscopic fields were analyzed per mouse for synapse number, which was counted manually from the red image only.

Ultrastructural imaging of tibialis nerve and gastrocnemius muscle was performed as described (Franco et al., 2020). Between 80 and 120 axon cross sectional areas were measured per mouse using National Institutes of Health Image J. The cross-sectional areas of the three wild-type control tibialis nerves were grouped together to establish quartile limits, and the individual data from all experimental animals were then analyzed according to those limits.

### Data Presentation and Statistical Analyses.

Most data are reported as means ± S.D. or S.E.M., as indicated; EC50 values are reported as mean value with 95% confidence intervals. Two-group comparisons used Student’s *t* test. Multiple group comparisons used one-way ANOVA, and time-course by treatment group and genotype comparisons used two-way ANOVA, each with Tukey’s posthoc test for individual statistical comparisons. All ANOVA tables are provided in Supplemental Tables 1–5, indexed to individual figures. *P* < 0.05 was considered significant.

## Results

### A Novel Piperine Derivative is a Potent Mitofusin Activator.

**Fig. 1.**
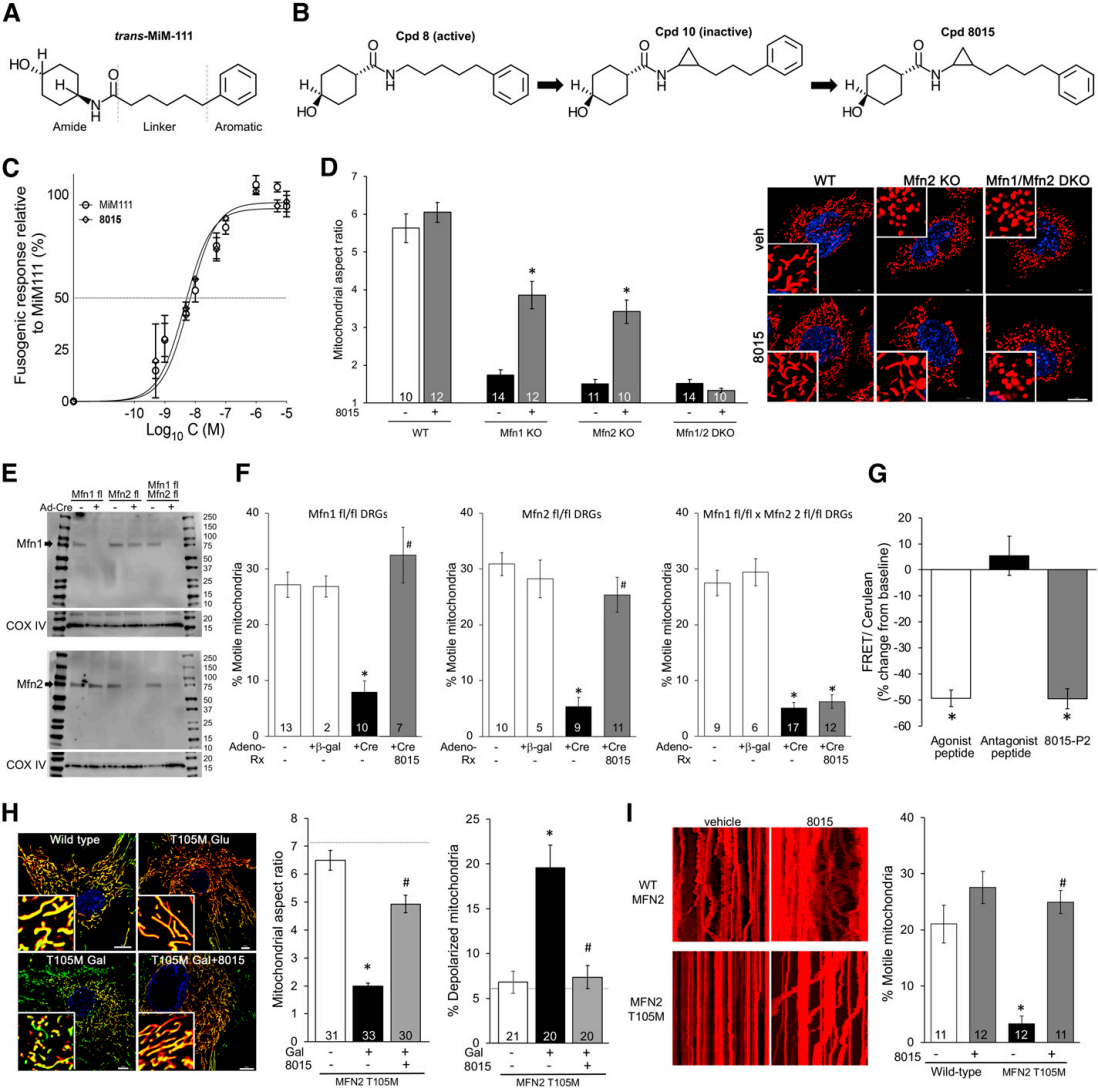
*Effects of 8015 on fibroblast and neuronal mitochondria.* (A) Chemical structure of *trans*-MiM111 as an exemplar of shared features for allosteric small molecule mitofusin activators. (B) Evolution of 8015 from compounds 8 and 10 described in Zhang et al., 2022. (C) Dose-response curves of 8015 and *trans*-MiM111 to induce mitochondrial elongation in Mfn2 null MEFs (means of three independent experiments). (D) Comparative fusogenic responses of 8015 (100 nM treated overnight) on MEFs derived from Mfn1, Mfn2, or Mfn1/Mfn2 double knockout mice; wild-type control data are shown for comparison. (E) Immunoblot analysis of in vitro mitofusin depletion by Cre-mediated recombination in cultured cells derived from *Mfn* floxed allele mice. (F) 8015 effects on mitochondrial motility in DRGs expressing different mitofusins. (G) FRET analysis (*n* = 9) of 8015-induced Mfn2 conformational changes compared with Mfn agonist and antagonist peptides. A decrease in FRET signal reflects separation of amino and carboxyl terminal fluorophores, i.e., an unfolded conformation. (H) Representative images (left) and group quantitative data (right) showing 8015-mediated increase in mitochondrial aspect ratio and polarization of mitochondria in galactose-fed human Mfn2 T105M CMT2A patient fibroblasts. – Gal (galactose) shows metabolically unstressed control data; WT cell values are shown as horizontal dashed lines. (I) Representative kymographs (left) and group quantitative data (right) showing 8015-mediated enhancement of mitochondrial motility in reprogrammed MFN2 T105M CMT2A patient motor neurons. Data are means ± S.E.M.; n values for all except panels C and G are at the base of bars and indicate number of individual cells in which 30–50 mitochondria per cell were examined in 3–4 independent experiments. * = *P* < 0.05 vs. wild-type (F, H, I) or baseline (G); # = *P* < 0.05 vs. +Cre (F) or 8015-untreated T105M (H, I) by 1-way ANOVA with Tukey’s test. Data in (D) were analyzed in the absence and presence of 8015 by *t* test; * = *P* < 0.05. ANOVA tables for all figures having multiple data sets are in the supplement.

All small molecule allosteric mitofusin activators described to date (Rocha et al., 2018; Dang et al., 2020, 2021; Zacharioudakis et al., 2022; Zhang et al., 2022) have the general chemical architecture exemplified by the prototype mitofusin activator, *trans*-MiM111: cycloalkyl-carboxamide and aromatic groups are separated by a linker moiety of defined length (Fig. 1A), thereby reproducing the physicochemical characteristics of amino acid side chains within the so-called mitofusin “zipper moiety” that contributes to peptide–peptide interactions driving mitofusin protein conformation (Rocha et al., 2018; Dorn, 2019). As the piperine derivatives previously described did not have in vivo pharmacokinetic or pharmacodynamic properties acceptable for a clinically useful drug, here we modified functionally inactive “Cpd 10” (Zhang et al., 2022) to optimize the spacing between its cycloalkyl-carboxamide and aromatic groups by adding one carbon to the linker chain (Supplemental Fig. 1). The resulting compound, (1r,4r)-4-hydroxy- N-[(1S,2S)-2-(4-phenylbutyl)cyclopropyl]cyclohexane-1-carboxamide, designated 8015 (M.W. 315.46) (Fig. 1B) (Dorn, 2023), was obtained at > 96% purity (Supplemental Fig. 2, A**–**C). 8015 activated mitofusins at a similar ∼5 nM potency as *trans*-MiM111 and other phenylhexanamides (Fig. 1C) (Dang et al., 2020, 2021). However, 8015 exhibited favorable pharmaceutical properties versus its phenylhexanamide comparators, including enhanced passive membrane permeability measured as parallel artificial membrane permeability, a longer plasma half-life after oral administration to mice, and high brain/plasma partitioning (Table 1).

**TABLE 1:** Comparative characteristics of 8015 vs. trans-MiM111 and CPR1-B

		***trans*-MiM111**	**CPR1-B**	**8015**
**Calculated Properties**				
**MW**		289.4	301.4	315.46
**calc logP**		3.22	3.09	3.57
** TPSA**		49.33A	49.33A	49.33A
**Functional Properties**				
**EC50 mito elongation**		7.7 nM	4.3 nM	5.3 nM
**In vitro DMPK**				
**Plasma protein % bound**	**H**	90.4%	94.4%	95.3%
	**M**	96.7%	95.5%	96.8%
**Aqueous solubility (pH 7.4)**		175 uM	<10 uM	30 uM
** Liver microsomes t1/2**	**H**	>145 min	>145 min	139 min
	**M**	126.9	114.1 min	193 min
** Parallel artificial membrane permeability (Pe, nm/s)**		22.29	58.45	86.8
** P-gp efflux ratio**		1.74	0.433	.99–1.1
**In vivo plasma PK (i.v.)**		10 mg/kg	10 mg/kg	10 mg/kg
** t1/2**		1.1 h	1.64 h	0.8 h
**In vivo plasma PK (p.o.)**		50 mg/kg	50 mg/kg	50 mg/kg
**t1/2**		1.2 h	1.67 h	2.2 h
** Bioavailability**		61%	59%	44%
** Brain/plasma ratio**		0.1	0.38	0.865

H, human; M, mouse.

*Trans*-MiM111 data are from Dang et al., 2020 (cpd 13b); CPR1-B data are from Dang et al., 2021 (cpd 5b).

Like phenylhexanamide mitofusin activators, 8015 improved mitochondrial fragmentation caused by ablation of either the *Mfn1* or *Mfn2* gene in MEFs but lacked fusogenic activity in cells deficient in both of its *Mfn* protein targets (Fig. 1D). Moreover, in what we believe to be the first time this metric has been reported, 8015 corrected mitochondrial dysmotility evoked by conditional ablation of either *Mfn1* or *Mfn2* (Fig. 1E) in mouse DRG neurons but had no effect on mitochondrial transport within processes of DRG neurons lacking both Mfn1 and Mfn2 (Fig. 1F; Supplemental Fig. 3). Thus, 8015 requires either Mfn1 or Mfn2 to enhance mitochondrial fusion and transport. Like previously described mitofusin activators (Dang et al., 2021), 8015 increased the probability that MFNs spend time in an open, fusion-permissive conformation (Fig. 1G).

To determine if 8015 would correct mitochondrial dysfunction in CMT2A we studied its actions on human CMT2A patient (MFN2 T105M mutation) cells. 8015 reversed hallmark mitochondrial fragmentation (a marker of impaired fusion) and loss of polarization (a correlated marker of impaired respiratory function) in metabolically stressed (galactose-fed; Dang et al.; 2022) CMT2A dermal fibroblasts (Fig. 1H).

Loss of mitochondrial motility in neuronal axons is widely accepted as a key event underlying neuronal die-back in CMT2A and other progressive neurodegenerative diseases (Knott et al., 2008; Chen and Chan, 2009; Sheng and Cai, 2012). Thus, the ability of a compound to restore mitochondrial motility can predict therapeutically beneficial interventions (Dorn, 2021; Dorn and Dang, 2022; Dang et al., 2023; Dorn, 2023). 8015 normalized mitochondrial dysmotility in reprogrammed motor neurons derived from the same CMT2A patient carrying MFN2 T105M (Fig. 1I).

**Fig. 2.**
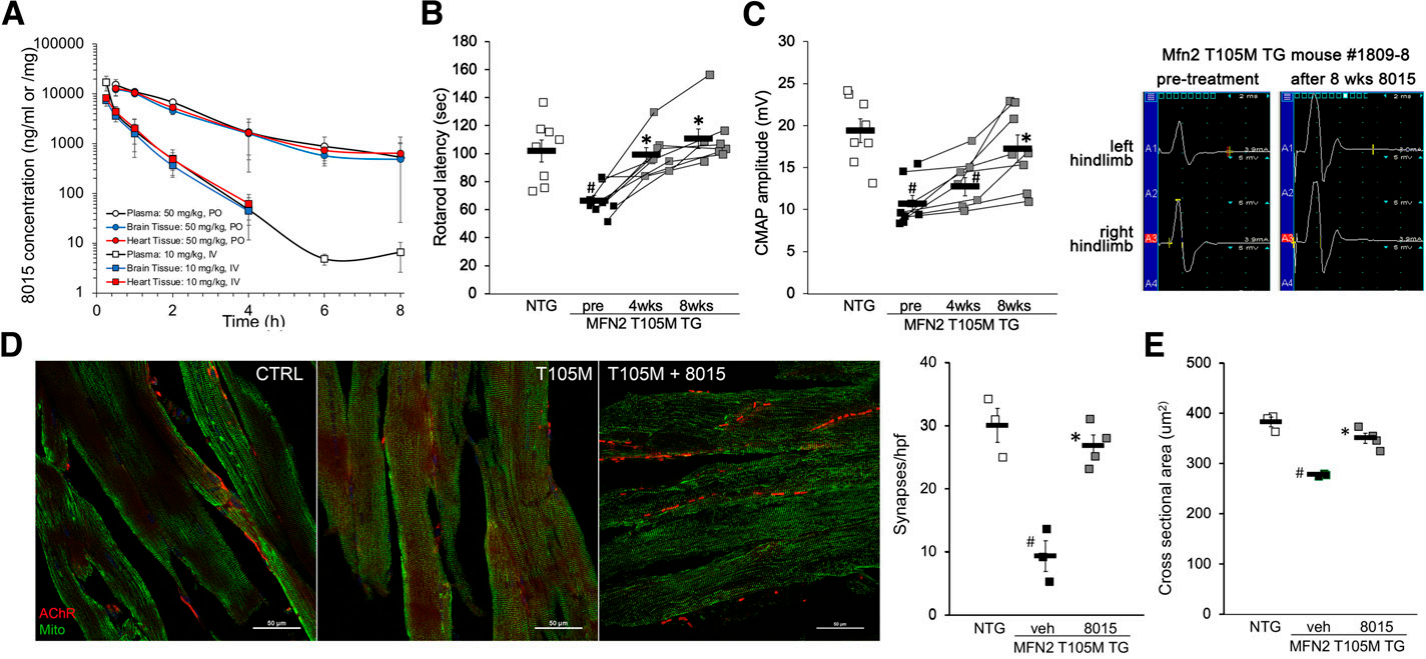
*8015 reverses CMT2A phenotypes in Mfn2 T105M motor neuron transgenic mice.* (A) 8015 plasma and tissue concentrations after 50 mg/kg oral (circles) or 10 mg/kg intravenous (squares) administration. White is plasma level, blue is brain level, red is heart level; *n* = 3 mice per dose. B–E. Effects of 8015 treatment (50 mg/kg/d orally) on neuromuscular function measured as rotarod (B) and neuroelectrophysiological CMAP amplitude (C); eight WT and eight Mfn2 T105M mice were studied. (D) Tibialis muscle neuronal synapse density (stained red in representative confocal micrographs) and (E) myocyte cross-sectional area; four mice per group underwent histological assessment. # = *P* < 0.05 vs. wild-type control; * = *P* < 0.05 vs. pretreatment (1-way ANOVA with Tukey’s pairwise comparison).

Short-acting *trans-*MiM111 for “burst” mitofusin activation and longer acting N-(trans-4-hydroxycyclohexyl)-2-(3-phenylpropyl)cyclopropane-1-carboxamide (CPR1-B) administered twice daily for “sustained” mitofusin activation reportedly reversed neuromuscular dysfunction in mice with motor neuron specific transgenic MFN2 T105M expression (Franco et al., 2020, 2022). We observed an in vivo plasma half-time for 8015 of 2.2 h after oral administration to mice, which modestly exceeds that reported for CPR1-B (Fig. 2A; Table 1). For proof-of-concept we tested 8015-mediated disease mitigation in the same transgenic CMT2A mouse model. As previously reported (Franco et al., 2020, 2022), fifty week-old MFN2 T105M transgenic mice exhibited a decrease in rotarod latency (the time a mouse can stay on an accelerating rotating cylinder) and neuroelectrophysiological compound muscle activation potential (CMAP) amplitude. Administration of 8015 (50 mg/kg once daily by oral gavage) to these mice normalized neuromuscular dysfunction (Fig. 2B) and neuroelectrophysiological abnormalities (Fig. 2C). 8015 treatment restored neuromuscular synapse density in distal hindlimb tibialis muscles (Fig. 2D) and normalized tibialis muscle myocyte cross sectional area (Fig. 2E). Thus, 8015 abrogates CMT2A neuromuscular phenotypes previously described in MFN2 T105M motor neuron transgenic mice.

### Compound 8015 Exhibits Stereoisomer-Specific Mitofusin Activator Activity.

**Fig. 3.**
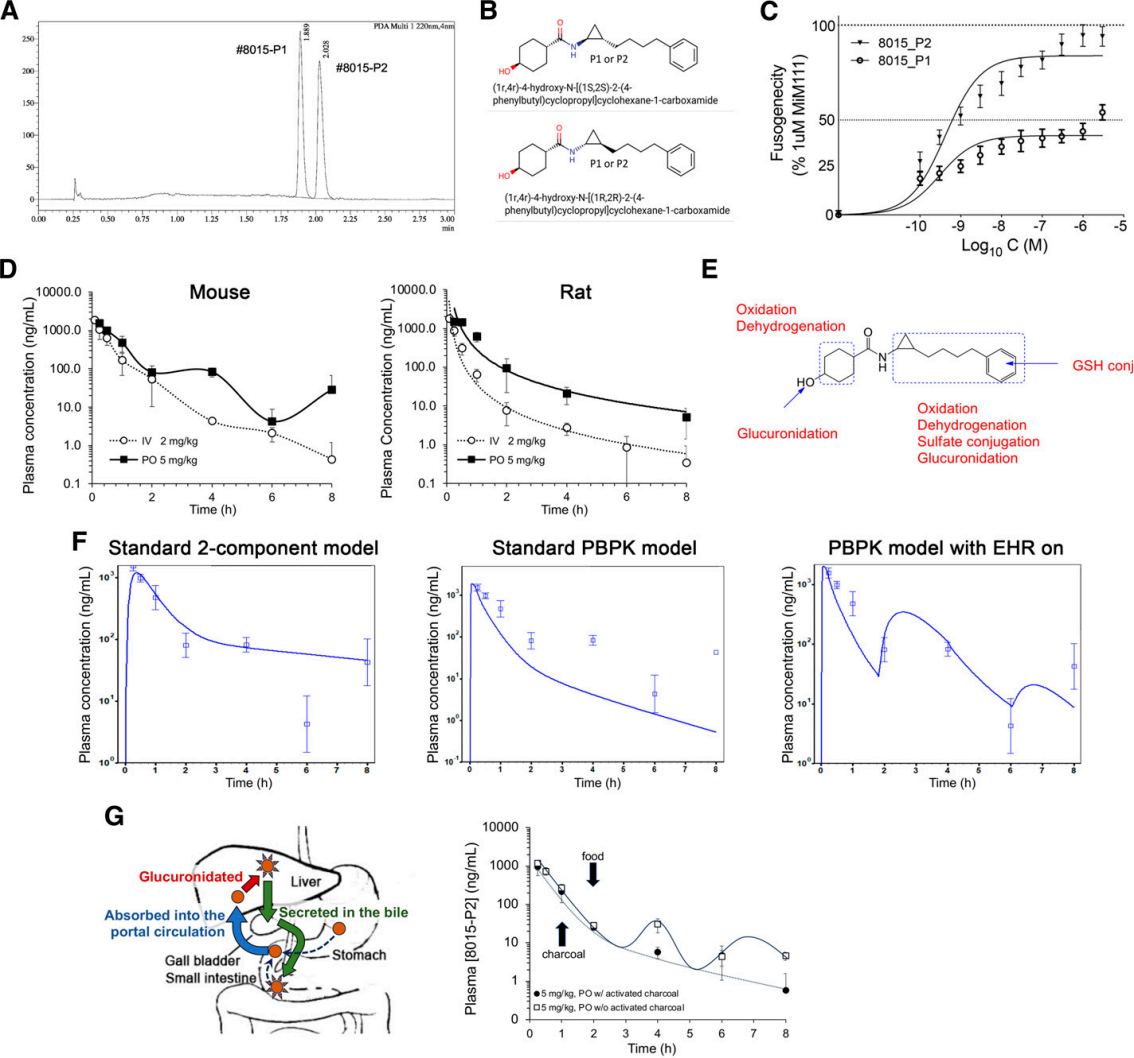
*Enterohepatic recirculation of the highly potent 8015 isomer, 8015-P2*. (A) Chiral separation of 8015 isomers (shown in B). (C) Stereospecific differences in fusogenic activity of the two 8015 isomers (*n* = 4 each). (D) Plasma pharmacokinetics of 8015-P2 in mice (left) and rats (right) after intravenous (white) or oral (black) administration at the doses indicated (*n* = 3 each). (E) Summary of major 8015-P2 metabolic events measured in cultured hepatocytes (full metabolic schemes are in Supplemental Fig. 4). (F) GastroPlus modeling of mouse PK data from (D) using different parameters. (G) 8015-P2 enterohepatic recirculation. (left) schematic depiction; (right) charcoal administration changes EHR fit (see panel F, right) to standard physiological based pharmacokinetic model (see panel F, middle) (*n* = 4 each).

8015 has two chiral centers, one at the cyclohexanol group and the other at the linker cyclopropyl group. Based on previously established stereochemistry/activity relationships that identified the *transcyclohexanol* as more active in phenylhexanamide mitofusin activators (Dang et al., 2020), 8015 was synthesized as *trans* at the cyclohexanol moiety. Supercritical fluid chromatography was employed to evaluate cyclopropyl isomer composition: two 8015 isoforms separated in ∼1:1 ratio (Fig. 3A), designated 8015-P1 (the faster eluting peak) and -P2 (the slower eluting peak). Because the chemical synthesis scheme (see Supplemental Fig. 1) produces only *trans-* at the cyclopropyl group, P1 and P2 represent the *trans-* isomers (Fig. 3B). Comparative analysis of their fusogenic potencies showed 8015-P2 to be markedly more potent and effective than 8015-P1 for inducing fusion of Mfn2-deficient mitochondria (Fig. 3C). Notably, 8015-P2 was ∼10-fold more potent as a fusogenic factor (EC50 = 623PM; 95% confidence limits 439 - 863PM, *n* = 4) than *trans*-MiM111 or CPR1-B (see Table 1).

As described above (see Fig. 2A and Table 1), the 8015 enantiomeric mixture exhibited a longer plasma elimination half-life when administered orally compared with intravenous injection. Moreover, we observed atypical oral 8015-P2 plasma pharmacokinetics in mice, in which periods of seemingly normal elimination were followed by phasic increases in plasma compound levels (Fig. 3D, left). This pharmacokinetic profile is characteristic of EHR of drugs, wherein the drug is glucuronidated in the liver, secreted in the bile, excreted periodically into the small intestine when the gall bladder empties, and de-glucuronidated by intestinal flora, thereby freeing the compound up for intestinal reabsorption (Taft, 2009). Indeed, waxing and waning of plasma 8015-P2 levels was not observed after oral administration to rats, which do not have gallbladders (Higashiyama et al., 2018) (Fig. 3D, right). Moreover, in vitro hepatocyte metabolism studies identified glucuronidation as a major metabolic pathway for 8015 (Fig. 3E, Supplemental Fig. 4) and GastroPlus modeling (Parrott et al., 2009) of mouse pharmacokinetics was most consistent with EHR (Fig. 3F). EHR of 8015-P2 was confirmed by repeating the mouse oral PK study without and with oral charcoal administration to interrupt intestinal–hepatic recirculation (Christophersen et al., 2002) (Fig. 3G, left). Administration of activated charcoal by gavage (2 mg/kg slurry) 1 hour after oral dosing of 5 mg/kg 8015-P2 produced the more standard physiological based pharmacokinetic drug elimination pattern (Fig. 3G, right), while decreasing t_1/2_ by 36% (1.21 hours vs. 1.88 h) and AUC_last_ by 17% (739 vs. 887 hours*ng/ml). Thus, 8015-P2 undergoes enterohepatic recirculation that increases its oral bioavailability. EHR has not been described for other mitofusin activators.

### *Mfn2 T105M and M376V Mutations Evoke Distinct in Vivo Neurologic Phenotypes*.

CMT2A-linked MFN2 mutations include T105M located within the GTPase domain (Pipis et al., 2020) and M376V located in the catalytically inactive coiled-coil first heptad repeat domain (Nightingale et al., 2014; Larrea et al., 2019). Mfn2 T105M and M376V knock-in mice were recently described in the context of a cardiomyopathy caused by the human MFN2 mutation R400Q (Franco et al., 2023); neurologic phenotypes of these mice were not reported.

**Fig. 4.**
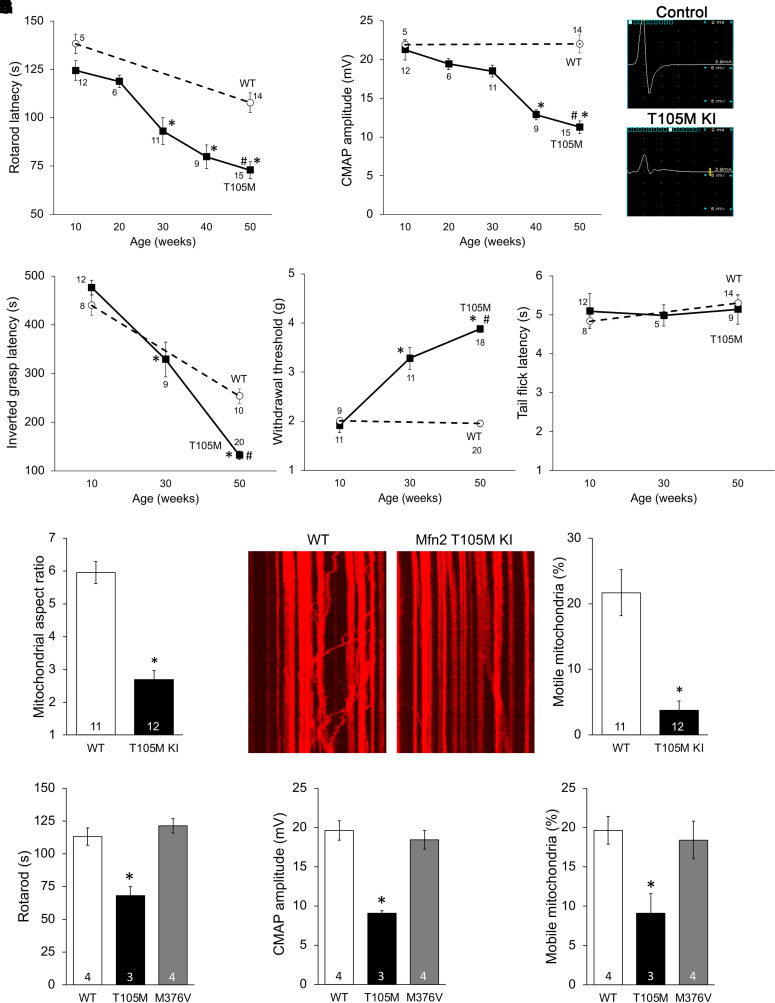
*Neurologic and mitochondrial phenotypes of heterozygous Mfn2 T105M KI mice*. (A, B) Age-dependent decrease in rotarod latency (A) and sciatic nerve CMAP amplitudes (B). Neuroelectrophysiological tracings from representative 50-week-old wild-type control and T105M KI mice are on the right. (C–E) Age-dependence of Mfn2 T105M KI mouse inverted grasp latency (C), von Frey testing of foot pad withdrawal threshold (D), and tail flick after immersion in hot water (E). F, G. Mitochondrial studies in cultured DRGs derived from Mfn2 T105M KI mice: mitochondrial aspect ratio (F); mitochondrial motility (G). Representative kymographs are to the left in (G). (H–J) Comparative studies of heterozygous 50-week-old Mfn2 T105M KI and homozygous 500-week-old Mfn2 M376V mice. Data are means ± S.E.M.; n values below markers or at base of bars indicate number of individual mice/cells studied per condition. A-E: * = *P* < 0.05 versus 10 weeks (1-way ANOVA); # = *P* < 0.05 versus WT (2-way ANOVA). F-J: * = *P* < 0.05 versus WT control (ANOVA or *t* test). Tukey’s test was used for all pairwise ANOVA comparisons.

Here, we characterized neurologic phenotypes of heterozygous Mfn2 T105M KI mice [homozygous Mfn2 T105M KI mice died before embryonic day (E) 12.5 (Franco et al., 2023)]. Compared with age-matched wild-type (WT) Mfn2 controls, neuromuscular function of Mfn2 T105M mice progressively declined over the first year of life, manifested as a decrease in rotarod latency (Fig. 4 A), neuroelectrophysiological CMAP amplitude (Fig. 4B), and the time to fall from an inverted grid (a measure of grip strength; Fig. 4C). Likewise, sensory neuron function measured as paw withdrawal in response to stimulation with a small filament (von Frey test) deteriorated over the same time course (Fig. 4D), whereas sensitivity to a thermal stimulus was unaffected by introduction of the CMT2A mutation (tail withdrawal from hot water, Fig. 4E). Phenotype progression was similar in male and female Mfn2 T105M KI mice (Supplemental Fig. 5). (Histological and ultrastructural features of Mfn2 T105M KI mice are presented in Fig. 6.) Moreover, cultured DRGs derived from Mfn2 T105M KI mice exhibited mitochondrial fragmentation (Fig. 4F) and dysmotility (Fig. 4G) characteristic of CMT2A.

In contrast to MFN2 T105M, M376 mutations have normal catalytic GTPase activity and, while lacking intrinsic fusogenic function, do not dominantly suppress mitochondrial fusion provoked by normal MFN2 (“functional null”; Franco et al., 2023). In comparison with homozygous Mfn2 T105M KI mice that die as embryos before E12.5 (Franco et al., 2023), homozygous Mfn2 M376V KI mice appeared healthy throughout embryogenesis and as adults. We compared neuromuscular and mitochondrial function of ∼50-week-old homozygous Mfn2 M376V KI to that of heterozygous Mfn2 T105M KI mice. For each parameter, M376V mice exhibited normal function (Fig. 4, H–J**).** The comparison of Mfn2 T105M GTPase domain and M376V non-GTPase domain mutant KI mice provides additional direct experimental support for the idea that MFN2 mutations provoking different types of mitochondrial dysfunction will induce distinct in vivo phenotypes, affording a mechanistic basis for phenotypic variability among CMT2A patients (Pipis et al., 2020).

### Mitofusin Activation with Oral 8015-P2 Reverses Mitochondrial Dysmotility in DRG Neurons and Sciatic Nerves of Mfn2 T105M Knock-in Mice.

**Fig. 5.**
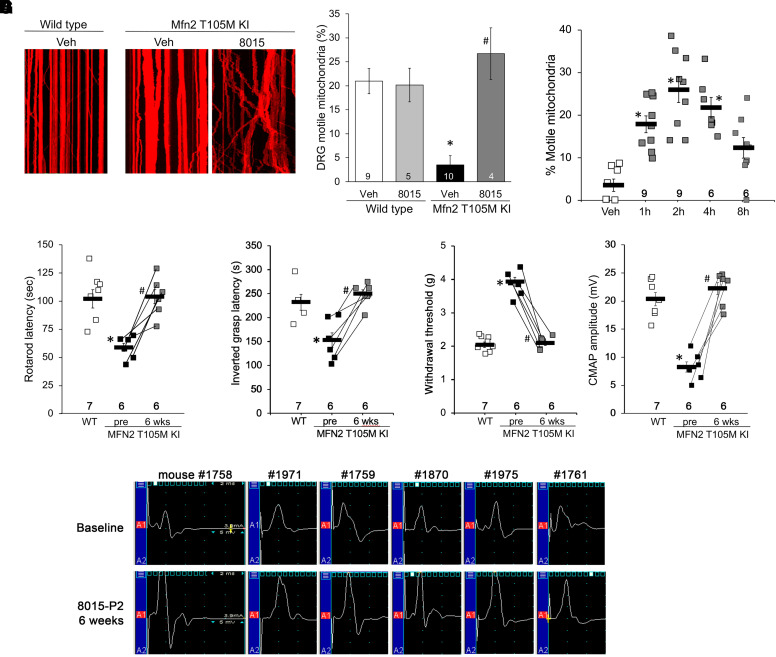
*8015-P2 reverses CMT2A phenotypes in Mfn2 T105M KI mice.* (A) Mitochondrial dysmotility in DRG neurons derived from Mfn2 T105M KI mice is reversed by culturing with 8015-P2 (100 nM, 48 hours). Representative kymographs are to the left; group quantitative data are on the right. (B) Time-dependent pharmacodynamic effects of orally administered 8015-P2 (100 mg/kg) on mitochondrial motility in Mfn2 T105M KI mouse sciatic nerve axons. (C–F) Improvement in Rotarod latency (C), inverted grasp (D), foot pad mechanical sensitivity (E) and CMAP amplitude (F) in Mfn2 T105M KI mice treated for 6 weeks with 8015-P2. G. Paired before and after 8015-P2 treatment CMAP tracings for all study KI mice. Number of mice studied per group is indicated in each panel. * = *P* < 0.05 vs. wild-type (A, C–F) or vehicle (B) control; # = *P* < 0.05 vs. pretreatment (ANOVA with Tukey’s pairwise comparisons).

We cultured sensory neurons from DRGs of Mfn2 T105M KI mice and visualized their mitochondria in live cell preparations after adenoviral transduction of mitochondria-targeted DS-Red2. Mitochondrial dysmotility in Mfn2 T105M KI DRG neuronal processes was corrected by addition of 8015-P2 (100 nM for 48 hours) (Fig. 5A). Likewise, administration of 8015-P2 (100 mg/kg by oral gavage) to Mfn2 T105M KI mice corrected mitochondrial dysmotility measured ex vivo in sciatic nerve axons (Fig. 5B). These results show that 8015-P2 corrects mitochondrial dysmotility caused by heterozygous expression of Mfn2 T105M, supporting the hypothesis that 8015-P2 could reverse neurologic phenotypes in Mfn2 T105M KI mice.

### 8015-P2 Reverses Motor and Sensory Deficits in Mfn2 T105M Knock-in Mice.

Mfn2 T105M KI mice of both sexes were aged until ∼50 weeks of age, at which time their baseline motor function (rotarod latency, inverted grip strength and neuro-electrophysiological CMAP) and sensory function (von Frey assay of foot pad mechanical sensitivity) were measured. The mice were then treated with 8015-P2, 50 mg/kg once daily by oral gavage for 6 weeks. 8015-P2 normalized motor neuron functional metrics (Fig. 5, C and D), a sensory neuron metric (Fig. 5E) and the typical CMT2A neuroelectrophysiological abnormality (Fig. 5, F and G).

### 8015-P2 Corrects Histological and Ultrastructural Defects in Mfn2 T105M Knock-in Mice.

**Fig. 6.**
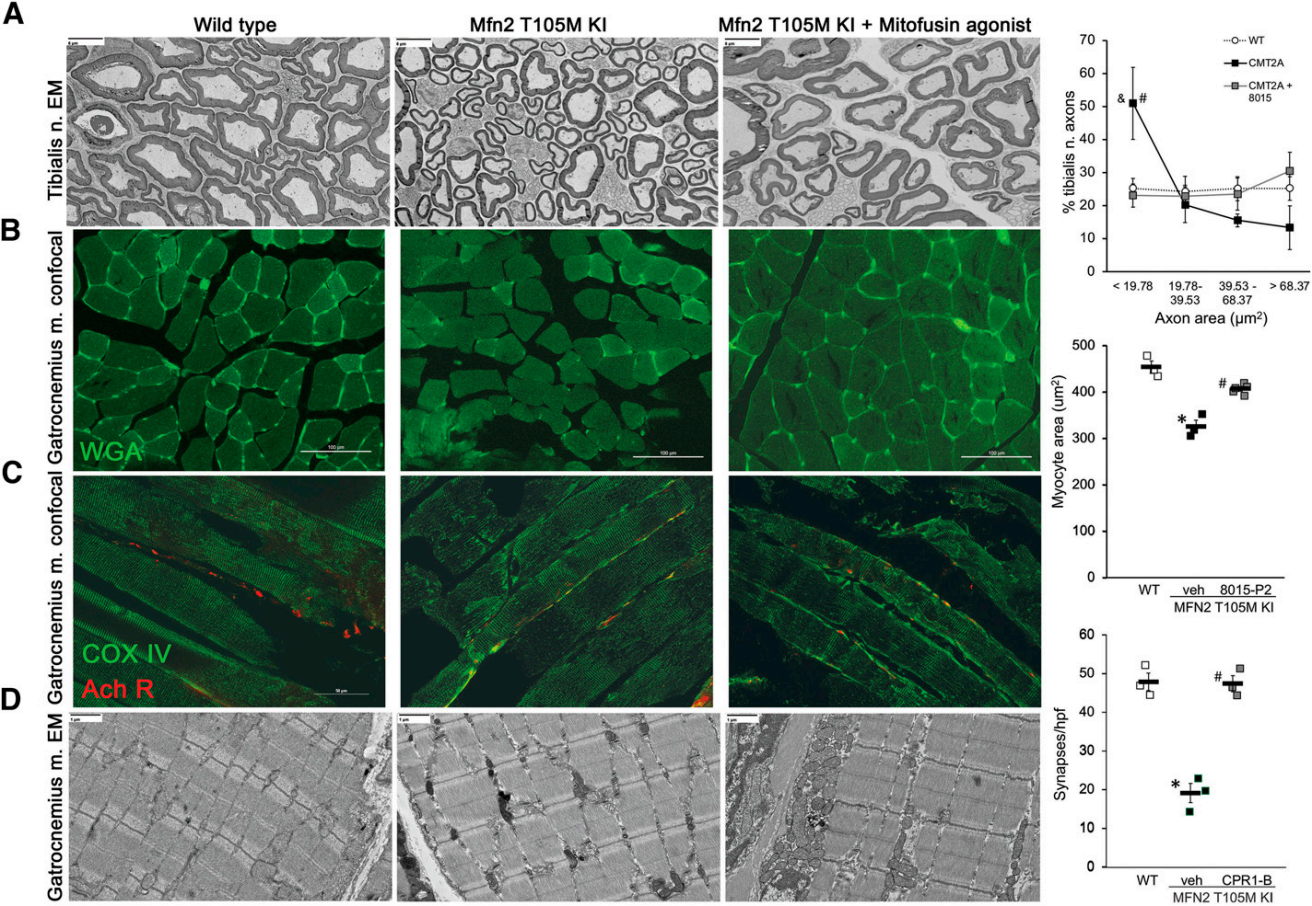
*Histological and ultrastructural findings in Mfn2 T105M KI mice and effects of mitofusin activation.* (A) Representative transmission electron micrographs (TEM; 400x) of tibialis nerves in cross section. Quantitative axon cross sectional area data separated into normal (WT) quartiles are to the right; CMT2A = Mfn2 T105M KI. (B) Representative fluorescein-labeled wheat germ agglutinin staining of gastrocnemius muscle showing myocyte cross sections. Quantitative data are to the right. Each marker represents mean value of ∼100 axon (A) or myocyte (B) determinations from a single mouse; *n* = 4 mice per group. (C) Gastrocnemius muscle myocyte acetyl choline receptor (Ach R) labeled neuromuscular synapse density measured as *α*-bungarotoxin staining (red); green mitochondria are labeled with anticytochrome oxidase (COX) intr; (*n* = 3 mice/group). (D) TEM (5,000x) of gastrocnemius muscles showing normal myofilament structure. * = *P* < 0.05 vs. WT; # = *P* < 0.05 vs. Mfn2 T105 KI+veh (ANOVA).

We found that reversal of sensory and motor neuron phenotypes correlated with improvement in histological and ultrastructural features typical of CMT2A. Consistent with neuronal regrowth, the increase in proportion of neurons with small axonal areas (lowest quartile) in tibialis nerves that innervate the lower hindlimb was reversed after 8015-P2 administration for 6 weeks; neuron myelination was normal in all groups (Fig. 6A). Likewise, the reduction in myocyte cross-sectional area in gastrocnemius muscles of the distal Mfn2 T105M KI mouse hind limb (Fig. 6B) and the decrease in neuromuscular synapse density (Fig. 6C) were reversed by mitofusin activation. Gastrocnemius myoatrophy appeared to be a consequence of neuronal die-back rather than a primary myopathy as transmission electron microscopy showed normal myofilament architecture in the Mfn2 T105M KI mice, which was not changed by mitofusin activation (Fig. 6D).

## Discussion

The current studies present two important new findings. First, we describe the prototype pharmaceutically acceptable member of a new chemical class of “reverse carboxamide” mitofusin activators, 8015-P2. This piperine-derived compound is ∼10-fold more potent and has greater tissue partitioning than previously-described mitofusin activators. Uniquely, 8015-P2 undergoes enterohepatic recirculation that improves oral bioavailability and prolongs in vivo exposure. In multiple in vitro and in vivo preclinical models, 8015-P2 reversed mitochondrial, cellular, and neuromuscular phenotypes caused by the GTPase-inactivating CMT2A mutation, MFN2 T105M. Although less advanced in development than phenylhexanamide mitofusin activators, such as *trans*-MiM111 and CPR1-B, 8015-P2 represents an attractive alternative compound should problems arise in the clinical development of what is a narrow pipeline of small molecule mitofusin activators.

Secondly, we describe sensory and motor neuron phenotypes for a mouse Mfn2 T105M knock-in model of CMT2A. The knock-in mouse phenotype recapitulated seminal features from the human condition permitting us to evaluate both neuromuscular (motor) and sensory neuron responses to 8015-P2. Our results suggest that mitofusin activation can benefit disease-related CMT2A pathology beyond just neuromuscular dysfunction.

It was recently reported that the natural product piperine, derived from black pepper, can activate mitofusins with approximately the same potency as phenylhexanamide compounds such as *trans*-MiM111 and CPR1-B that improved neuromuscular degeneration in motor neuron-specific MFN2 T105M transgenic mice (Franco et al., 2020, 2022; Zhang et al., 2022). Because piperine has many diverse biological targets and can be cytotoxic at higher concentrations (Piyachaturawat et al., 1983; Bhardwaj et al., 2002; Lee et al., 2005; McNamara et al., 2005), we modified its chemical structure to achieve greater specificity for mitofusins (Zhang et al., 2022). The resulting compound, designated 8015, is chemically similar to CPR1-B as they share *transcyclohexyl* groups connected to phenyl groups by a carboxamide linker having a cyclopropyl ring. The major difference is the orientation of the carboxamide moiety, which likely contributes to distinct pharmaceutical properties. The convergence of chemical structures for these two compounds is based in part on the application of knowledge gained from studying the relationship between chemical structure and fusogenic activity during the rational design phase of first and second generation small molecule mitofusin activators (Rocha et al., 2018; Dang et al., 2020, 2021). The possibility that the amide moiety could be modified while preserving mitofusin activating activity was suggested by the piperidine structure of piperine (Zhang et al., 2022). It was gratifying that reversing the carboxamide orientation and adjusting the linker length by a single carbon evoked a 10-fold increase in fusogenic potency and raised tissue/plasma partitioning to near unity. From a pharmaceutical perspective, the most impactful characteristic of 8015-P2 may be EHR. EHR is well recognized for drugs, such as the nonsteroidal anti-inflammatory agent indomethacin (Duggan et al., 1975; Maseda and Ricciotti, 2020), that are excreted in bile after initial absorption from the gastrointestinal tract and delivered to the liver via the portal circulation. A positive aspect of drug EHR is extended plasma half-life and greater oral bioavailability, as observed for 8015-P2. EHR can also be responsible for interspecies differences in drug response and toxicity that may confound preclinical evaluation and development. In a clinical context, levels of drugs subject to EHR can be affected by changes in intestinal motility, the quantity and quality of intestinal flora, and by gall bladder function. Additional pharmacokinetic and toxicological studies in nonrodent species will better inform these considerations and the candidacy of 8015-P2 for clinical introduction.

Including the present study, three different small molecular mitofusin activators (*trans*-MiM111, CPR1-B, 8015) have improved neuromuscular function in the mouse model of CMT2A provoked by motor neuron-specific transgenic expression of human MFN2 T105M (Franco et al., 2020, 2022). Each of these mitofusin activators rapidly (∼4 weeks) improved, and ultimately (6–8 weeks) normalized, results of Rotarod and neuroelectrophysiological testing. Based on these data, we anticipate that allosteric mitofusin activation by any compound that achieves acceptable- levels in the plasma and nervous system and is delivered in adequate amounts to neuronal tissues should prove beneficial in this syndrome. Moreover, based on the ability of multiple mitofusin activators to correct mitochondrial abnormalities of metabolically stressed primary dermal fibroblasts from CMT2A patients having a variety of MFN2 mutations (Dang et al., 2022; current study), it seems likely that mitofusin activation can correct CMT2A caused by most autosomal dominant loss-of-function mitofusin mutations. Thus, by activating normal endogenous MFN1 and MFN2, allosteric mitofusin activation is largely agnostic to the particular CMT2A causal MFN2 mutation.

Charcot-Marie-Tooth disease is the clinical descriptor used for a group of peripheral sensory-motor neuropathies exhibiting diverse presentations caused by a multitude of different genetic abnormalities (Fridman et al., 2015). CMT2A is distinguished from other forms of CMT by causal MFN2 gene mutations (Zuchner et al., 2004) and, in many instances, childhood onset and progression. Thus, Feely et al. reported an average age of symptomatic onset for CMT2A of 4.4 years and typical loss of ambulation by an age of 20 years (Feely et al., 2011). The majority of CMT2A-causing MFN2 mutations, including MFN2 T105M, are located within the amino terminal GTPase domain and are associated with earlier disease onset and a more aggressive clinical course, contrasting with CMT2A patients carrying mutations within the MFN2 carboxyl tail who exhibit a later age of onset and less severe neuromuscular signs (Feely et al., 2011; Stuppia et al., 2015). The classic signs of CMT2A include neurogenic atrophy of distal limb muscles and diminished sensation in the hands and feet, thought to result from loss of distal sensory and motor nerve function in the extremities (Bombelli et al., 2014). However, atypical laryngeal paralysis, retinal degeneration, and sensorineural hearing loss (Chung et al., 2006; Verhoeven et al., 2006; Züchner et al., 2006; Nicholson et al., 2008; Feely et al., 2011) suggest more general involvement within the nervous system. Although not measured in the present study, it will be interesting to see if rare retinal or auditory abnormalities reported in clinical CMT2A are also manifested in Mfn2 T105M KI mice.

The Mfn2 T105M KI mouse phenotype recapitulates typical clinical features of CMT2A. Young mice appear functionally normal, but between the age of 30–40 weeks develop progressive dysfunction of limb sensory (von Frey test) and motor (inverted grip, rotarod testing) nerves. In human patients, CMT2A severity can be followed over time using neuroelectrophysiological testing that detects characteristic loss of amplitude for compound motor activation potentials in the context of normal nerve conduction velocity (Harding and Thomas, 1980; Berciano et al., 2017). Likewise, Mfn2 T105M KI mice aged 30–40 weeks showed decreased CMAP amplitudes with normal conduction times. Loss of mitochondrial transport was fully evident in 8- to 12-week-old Mfn2 T105M mice (see Fig. 5B) and, in combination with mitochondrial damage from impaired fusion, resulted in delayed neuronal die-back (Fig. 6C) and emergence of sensory motor dysfunction (Fig. 4). This pathological sequence may explain why phenotypes of human and mouse CMT2A provoked by MFN2 T105M do not relate to specific developmental stages (e.g., child/adolescent/adult). Rather, disease phenotypes follow the time required to manifest sensory-motor dysfunction after a proportionally similar degree of nerve die-back.

Motor impairment in CMT2A patients tends to be more severe than sensory impairment, but it was still important to determine whether mitofusin activation can improve loss of sensation in this condition. Previous studies did not address this because the MFN2 T105M transgene used in the CMT2A mouse model was, in most instances, under control of the *MNX-1* (motor neuron and pancreas homeobox 1) gene promoter, providing for mutant MFN2 expression predominantly in motor neurons. As the human and mouse *MFN2* genes are highly homologous, differing in only 24 of 757 amino acids (with absolute sequence identity from AA 65–136), we created a more relevant CMT2A mouse model by introducing the T105M mutation into the mouse *Mfn2* gene. Strikingly, homozygous Mfn2 T105M mice died in utero before embryonic day 12.5 (Franco et al., 2023), consistent with absence of any reported homozygous MFN2 T105M human subjects.

Whereas our heterozygous T105M knock-in mice reproduced major clinical CMT2A phenotypes, both heterozygous and homozygous M376V mice had normal neuromuscular and sensory function. MFN2 M376V is a rare CMT2A mutation described in an elderly male with chronic and slowly progressive bilateral lower limb weakness with onset at age 11 (Nightingale et al., 2014; Larrea et al., 2019). Our recent characterization of cellular dysfunction caused by this mutation (Franco et al., 2023) showed loss of fusogenic activity without the dominant inhibition of mitochondrial fusion that is characteristic of most validated CMT2A mutations. In contrast with MFN2 T105M, M376 mutations do not negatively impact either mitochondrial respiration or mitochondrial motility (Franco et al., 2023). We posit this is because the underlying mechanism of mutational dysfunction for M376 mutations is impaired conformational shifting, rather than GTPase catalytic inactivity.

Efforts by our group and others to develop and advance a mechanistically-based disease-modifying treatment of CMT2A will need to overcome formidable scientific and regulatory obstacles faced by any new class of therapeutic compounds. Unanswered questions include: What is the potential toxicity of chronic systemic mitofusin activation? What are the pharmacokinetics of mitofusin activators in human beings and how might they be influenced by EHR for those compounds so impacted? What are optimal formulations for preclinical toxicity studies and for clinical applications? And, what is the proper therapeutic dose range and regimen for mitofusin activation in CMT2A? The final question might best be answered with a robust nonmouse model of CMT2A in which FDA-acceptable pharmacokinetic, pharmacodynamic, toxicity, and disease mitigation studies could be performed in the same species; such a model has not been reported.

## Abbreviations

8015: (1r,4r)-4-hydroxy- N-[(1S,2S)-2-(4-phenylbutyl)cyclopropyl]cyclohexane-1-carboxamide
8015-P2: The slower eluting peak of 8015 on chiral SFC
CMAP: compound muscle action potential
CMT: Charcot-Marie-Tooth disease
CPR1-B: N-(*trans*-4-hydroxycyclohexyl)-2-(3-phenylpropyl)cyclopropane-1-carboxamide
DRG: dorsal root ganglion
EHR: enterohepatic recirculation
FRET: Förster resonance energy transfer
KI: knock-in, i.e.. a CRISPR/Cas9-engineered genomic mutation
MEF: mouse embryonic fibroblast
MFN/Mfn: human/mouse mitofusin
PK: pharmacokinetic
*trans*-MiM111: N-(*trans*-4-hydroxycyclohexyl)-6-phenylhexanamide
WT: wild-type
